# Treatment outcome and its predictors among patients with status epilepticus in Africa: A systematic review and meta-analysis

**DOI:** 10.1016/j.rcsop.2026.100738

**Published:** 2026-04-02

**Authors:** Gebremariam Wulie Geremew, Yilkal Abebaw Wassie, Tirsit Ketsela Zeleke, Rahel Belete Abebe, Gebresilassie Tadesse, Setegn Fentahun, Sisay Sitotaw Anberbr, Habtamu Semagne Ayele, Assefa Kebad Mengesha, Demis Getachew, Alemante Tafese Beyna, Minichil Chanie Worku, Abaynesh Fentahun Bekalu, Masho Tigabe Tekle, Tekletsadik Tekleslassie Alemayehu

**Affiliations:** a Department of Clinical Pharmacy, School of Pharmacy, College of Medicine and Health Sciences, University of Gondar, Gondar, Ethiopia; b Department of Social and administrative Pharmacy, School of Pharmacy, College of Medicine and Health Sciences, University of Gondar, Gondar, Ethiopia; c Department of Psychiatry, School of Medicine, College of Medicine and Health Sciences, University of Gondar, Gondar, Ethiopia; d Department of Pharmacology, School of Pharmacy, College of Medicine and Health Sciences, University of Gondar, Gondar, Ethiopia; e Department of Medical Nursing, School of Nursing, College of Medicine and Health Sciences, University of Gondar, Gondar, Ethiopia; f Department of Pharmacy, College of Health Sciences, Debre Markos University, Debre Markos, Ethiopia; g Department of Pharmaceutical Chemistry, School of Pharmacy, College of Medicine and Health Sciences, University of Gondar, Gondar, Ethiopia

**Keywords:** Treatment outcome, Predictors, Status epilepticus, Systematic review and meta-analysis

## Abstract

**Background:**

Status epilepticus (SE) is a life-threatening emergency that requires urgent care to prevent serious complications or death. Despite treatment advances, mortality rates remain high in Africa due to various risk factors. This review aims to evaluate treatment outcomes and their predictors among SE patients in Africa.

**Methods:**

A comprehensive literature search was conducted using various databases including African Journals Online, Hinari, Google Scholar, PubMed, Science Direct, EMBASE, Cochrane Database, Sci-Hub, and Scopus. The quality of the included studies was assessed using the Newcastle–Ottawa Scale (NOS) checklist. Data on study characteristics and prevalence estimates were pooled using a random-effect meta-analysis, with additional subgroup and sensitivity analyses conducted. Potential publication bias was evaluated through both visual inspection and statistical methods.

**Results:**

This review included 10 studies and found that the overall prevalence of mortality and neurological sequelae among SE patients in Africa was 14.67% (95% CI: 7.70–21.64) and 19.82% (95% CI: 13.01–26.63), respectively. However, significant heterogeneity was observed, influenced by factors such as geographic region, study design, sample size, and patient age. Subgroup analyses indicated that the highest mortality prevalence was reported in Western Africa at 24.61% (95% CI: 22.72–26.49), in cross-sectional studies at 17.8% (95% CI: 3.9–31.7), and among adult populations at 21.02% (95% CI: 15.30–26.74). Meta-regression analysis revealed a positive association between sample size and the log odds of SE (coefficient = 0.009, *p* = 0.004). Furthermore, mortality was significantly associated with hypoglycemia [OR = 5.06 (95% CI: 2.65–9.65)], bacterial meningitis [OR = 3.18 (95% CI: 1.65–6.12)], and inadequate treatment [OR = 6.29 (95% CI: 2.66–14.88)].

**Conclusion:**

Our findings demonstrate a significant burden of mortality and neurological sequelae associated with status epilepticus, particularly in Western Africa and the adult population. Hypoglycemia, bacterial meningitis, and inadequate treatment were identified as independent predictors of mortality in patients with status epilepticus.

## Highlights

• Mortality among status epilepticus (SE) patients in Africa is 14.67%, with neurological sequelae at 19.82%.
• Western Africa reported the highest mortality (24.61%) compared to other regions.
• Adults experienced higher mortality (21.02%) than children.
• Key predictors of mortality include hypoglycemia, bacterial meningitis, and inadequate treatment.
• Significant heterogeneity observed across studies, influenced by region, design, and patient demographics.
• Urgent improvement in SE management and prevention strategies is critical to reducing mortality in Africa.

## 1 Introduction

Status epilepticus (SE) is a neurological emergency that requires immediate evaluation and treatment to prevent severe complications or death. Traditionally, it was defined as a seizure lasting 30 min or longer or as multiple seizures without the patient regaining full consciousness between episodes. However, current guidelines from organizations like the Neurocritical Care Society and the International League Against Epilepsy (ILAE), identify status epilepticus as continuous clinical and/or electrographic seizure activity lasting 5 min or more, or as recurrent seizures without return to baseline mental status between events.^1^, ^2^, ^3^ In this review, we adopt the modern 5-min definition of SE, as recommended by the ILAE and contemporary clinical guideline, as it best reflects current clinical practice and aligns with early treatment principles. However, we acknowledge that several studies conducted in African settings, particularly older or resource-limited investigations employed the traditional 30-min definition. To ensure a comprehensive synthesis of available regional evidence, studies using both definitions were included. SE can present in various forms, including convulsive, non-convulsive, focal motor, and myoclonic, and any type can progress to a refractory state. Convulsive status epilepticus is characterized by generalized tonic-clonic movements accompanied by impaired consciousness. Non-convulsive status epilepticus is defined by ongoing seizure activity detected on electroencephalogram (EEG) without visible tonic-clonic movements. Focal motor status epilepticus involves persistent, repetitive motor activity in a limb or muscle group, typically on one side of the body, and may occur with or without altered consciousness. Myoclonic status epilepticus presents as repeated myoclonic jerks and may also involve impaired awareness. Refractory status epilepticus occurs when seizures, either convulsive or non-convulsive, persist despite the administration of appropriate antiseizure medications.^4^ SE is the most common pediatric neurological emergency.^5^, ^6^

The annual incidence of SE per 100,000 population varies across regions: 9.9 in French-speaking Switzerland,^7^ 13.1 to 16.5 in Italy,^8^, ^9^ 15.8 in Germany,^10^ 18.3 in Rochester, Minnesota,^11^ and as high as 41 in Richmond, Virginia.^12^ Case fatality rates for status epilepticus range from 7% to 39%.^7^, ^10^, ^13^, ^14^, ^15^, ^16^, ^17^, ^18^ A systematic review conducted in a developed country reported mortality rates for status epilepticus (SE) of 15.9% in adults, 13.0% across all age groups, and 3.6% in pediatric populations.^19^ However, comprehensive data on SE-related mortality in Africa remain limited, and the true incidence of SE in Sub-Saharan Africa is still not well established. Notably, a recent study from rural Kenya reported SE rates up to eight times higher than those documented in a comparable study conducted in the UK.^20^, ^21^

The causes of SE vary significantly across different patient populations, influenced largely by age and an individual's history of epilepsy. In patients with known diagnoses of epilepsy, the most frequent triggers are poor medication adherence, chronic intake of alcohol, irregular antiepileptic treatment and abrupt drug withdrawal.^15^, ^22^, ^23^ Conversely, in those without a prior history of epilepsy, common causes include metabolic disturbances, cardiovascular disease (CVDs), electrolyte imbalances, systemic infections, head injuries, central nervous system (CNS) infections, and toxic exposures.^23^ Etiology plays a critical role in determining outcomes, as highlighted by several epidemiological studies and clinical reports. Prompt identification of the underlying cause can significantly reduce neurological complications and mortality. Moreover, the International League against Epilepsy (ILAE) has broadened the etiological classification, recognizing that SE may be triggered by identifiable disorders of genetic, metabolic, structural, infectious, or toxic origin.^22^, ^24^

Africa faces a distinct set of healthcare and epidemiological challenges that substantially influence the management and outcomes of status epilepticus (SE). In addition to limited access to diagnostic tools such as electroencephalography and neuroimaging, many health systems experience delayed treatment initiation, inconsistent availability of antiseizure medications, and a critical shortage of trained neurologists and emergency care specialists.^15^, ^25^ These constraints are often compounded by delayed presentation to healthcare facilities, particularly in rural and underserved communities, where traditional beliefs, transportation barriers, and financial limitations may impede timely care. A notable consequence of these diagnostic limitations is the likely under-recognition of non-convulsive status epilepticus (NCSE), which lacks overt motor manifestations and requires EEG for confirmation. As a result, NCSE is probably substantially underdiagnosed in African settings, leading to underestimation of overall SE incidence, mortality, and morbidity, and biasing reported etiological distributions toward more clinically obvious convulsive presentations.

Furthermore, the epidemiological profile of SE in Africa differs markedly from that of high-income settings. Central nervous system infections, cerebral malaria, perinatal complications, traumatic brain injury, HIV-related neurological disorders, and malnutrition are more prevalent and represent major precipitating factors for SE across the continent.^26^, ^27^ These conditions not only increase the incidence of SE but also contribute to more severe clinical presentations and poorer outcomes. Consequently, the predictors of morbidity and mortality associated with SE in Africa are likely to differ from those identified in resource-rich regions.

Despite the growing burden of epilepsy and SE in Africa, region-specific data on treatment outcomes and their determinants remain limited. Most existing evidence guiding SE management is derived from high-income countries, where healthcare infrastructure, disease etiology, and patient populations differ substantially. The direct application of such data to African settings may therefore be inappropriate and risks overlooking context-specific risk factors that critically influence outcomes.

This review seeks to address this important knowledge gap by synthesizing available evidence on treatment outcomes and their predictors among patients with SE across Africa. By consolidating findings from existing studies, the review aims to provide a clearer understanding of regional mortality rates, morbidity patterns, and key clinical and health-system factors associated with adverse outcomes. These insights will support clinicians in identifying high-risk patients, refining clinical decision-making, and optimizing management strategies in resource-limited settings. Additionally, the findings will inform policymakers and stakeholders by highlighting gaps in care and guiding the development of context-appropriate, evidence-based protocols.

## 2 Method

### 2.1 Protocol registration, reporting, and search strategy

The main objective of this review was to determine the prevalence of mortality and identify its predictors among patients with status epilepticus (SE) in Africa. This review was prospectively registered in the International Prospective Register of Systematic Reviews (PROSPERO) under registration number CRD420251102193. The systematic review and meta-analysis were conducted and reported following the Preferred Reporting Items for Systematic Reviews and Meta-Analyses (PRISMA) guidelines for observational studies^28^ (see Table S1).

An extensive electronic literature search was conducted by two authors (GWG and TTA) for studies published up to June 2025. The search period covered April to June 2025, to include the most recent evidence on status epilepticus (SE) in African populations. Databases searched included PubMed, Science Direct, EMBASE, the Cochrane Library, Scopus, Hinari, and Google Scholar. The search strategy used a combination of Medical Subject Headings (MeSH) and free-text terms as follows: (“treatment outcome” OR fatality OR death OR prognosis OR outcomes) AND (predictors OR factors OR “associated factors” OR determinants) AND (“status epilepticus” OR SE) AND Africa (see Table S2). Additionally, the reference lists of all included studies were manually reviewed (snowballing technique) to identify further relevant articles not captured through the database search.

Data collection process, items and extraction.

GWG, TTA, and YAW were responsible for collecting the relevant studies. Search results from different databases were combined, and duplicate articles were removed using EndNote reference management software (version X7.2). Data extraction was independently carried out by two reviewers (GWG and TTA) using a standardized checklist developed in Microsoft Excel (Table S3). For the first outcome (magnitude), the checklist included details such as author name, publication year, country, study design, study population, sample size, and outcome measures. For the second outcome, which involved factors associated with the outcome, log odds ratios were computed from the original study data using two-by-two tables. Any discrepancies between the two reviewers were resolved through discussion, and when necessary, a third or fourth reviewer (GT or SF) was consulted. GWG and TKZ oversaw the entire data extraction and synthesis process.

### 2.2 Eligibility criteria

All included studies were conducted in African settings, and only facility-based observational study designs were considered. The study populations comprised both children and adults. Both published and unpublished articles that reported treatment outcomes and their associated determinants among children with status epilepticus were eligible for inclusion. Moreover, gray literature was incorporated to minimize publication bias. Although the database searches were limited to articles published in English.

The exclusion criteria encompassed studies lacking clearly defined primary outcomes, as well as systematic reviews, meta-analyses, editorials, and commentaries. This strategy ensured the inclusion of high-quality, peer-reviewed research that directly informed the understanding of treatment outcomes and risk factors associated with status epilepticus in the target population.

To address missing data, the following approaches were employed:

• Studies were excluded if more than 20% of key outcome or explanatory variable data were missing and could not be derived from the available information. Only studies reporting sufficient data to calculate the required effect estimates were included in the quantitative synthesis. No statistical imputation methods (e.g., mean substitution) were applied to handle missing data.

### 2.3 Outcome measurement

This systematic review and meta-analysis focused on estimating the pooled prevalence of mortality and identifying its associated predictors among patients with status epilepticus (SE) in Africa. The primary outcome—mortality—was measured based on data directly reported in the included studies. These studies typically defined mortality as death occuring either during the acute episode of SE, within a specified follow-up period, or as part of in-hospital outcomes following SE treatment.

In addition to studies where mortality was the primary endpoint, the review also included studies in which mortality was reported as an explanatory variable affecting other clinical outcomes. For example, in some studies, mortality was used as a secondary measure to assess the impact of treatment delays, comorbidities, or sociodemographic factors on the prognosis of patients with SE. Mortality data were collected through patient records, clinical follow-up, or surveillance systems in both inpatient and outpatient settings.

### 2.4 Data analysis and synthesis

In the results section of this review, we provide a concise yet comprehensive summary of the statistical analyses performed on the included studies. We identified a range of clinical, demographic, and treatment-related factors associated with status epilepticus (SE), pooled effect estimates where appropriate, and assessed heterogeneity to better understand both the magnitude and direction of these associations.

Specifically, the factors analyzed included age, fever, etiology of SE (such as infectious, metabolic, or structural causes), duration of seizure before hospital presentation, prior history of epilepsy, delay in treatment initiation, non-adherence, presence of comorbidities, acidosis, level of consciousness at admission, and access to intensive care support.

For any factor with effect estimates available from at least two studies, a meta-analysis was conducted using the raw aggregated data reported in the original articles. Effect estimates are presented as odds ratios (ORs) with corresponding 95% confidence intervals (CIs) for each included study. Data were initially compiled in Microsoft Excel 2019 and then exported to STATA version 14 for statistical analysis.

To assess the consistency of findings across studies, we calculated heterogeneity using Cochran's Q test (chi-square statistic) and the I^2^ statistic, which were visualized in forest plots. An I^2^ value of 0% indicated no observed heterogeneity, while values of 25%, 50%, and 75% were interpreted as low, moderate, and high heterogeneity, respectively.^29^ A *p*-value of less than 0.05 was considered statistically significant. To ensure uniformity and comparability across studies, we adjusted effect estimates for certain factors by re-categorizing reference groups, allowing for consistent interpretation of associations with SE. For continuous variables, we presented separate pooled estimates when such data were available from multiple studies.

Given the substantial heterogeneity among the included studies, we employed the DerSimonian and Laird random-effects model to compute pooled effect sizes and corresponding I^2^ statistics. This approach accounts for both within-study and between-study variability, thus providing a more robust estimate of the overall effect.^30^ To test the null hypothesis, that there is no association between a given factor and treatment outcomes in SE patients, we calculated *p*-values using the standardized normal (z) statistic, with statistical significance set at *p* < 0.05.

To control for the risk of Type I error due to multiple comparisons, we applied the Benjamini–Hochberg procedure to regulate the false discovery rate.^30^ Potential publication bias was assessed through visual inspection of funnel plot asymmetry and statistically tested using Egger's weighted regression method. A *p*-value less than 0.05 in Egger's test was considered indicative of significant publication bias.

### 2.5 Subgroup analysis

We subsequently conducted additional subgroup analyses and meta-regression to explore whether key study characteristics influenced the observed associations. Specifically, we examined the effects of age group (pediatric, adult, or all ages), study design, the definition of status epilepticus (SE) used, and geographic location within Africa (North, East, South, West, and Central). Meta-regression was used to evaluate how these variables contributed to changes in *p*-values, thereby helping to identify potential sources of heterogeneity and better understand the variation in effect estimates across studies.

## 3 Results

### 3.1 Literature search and study selection

**Fig. 1:**
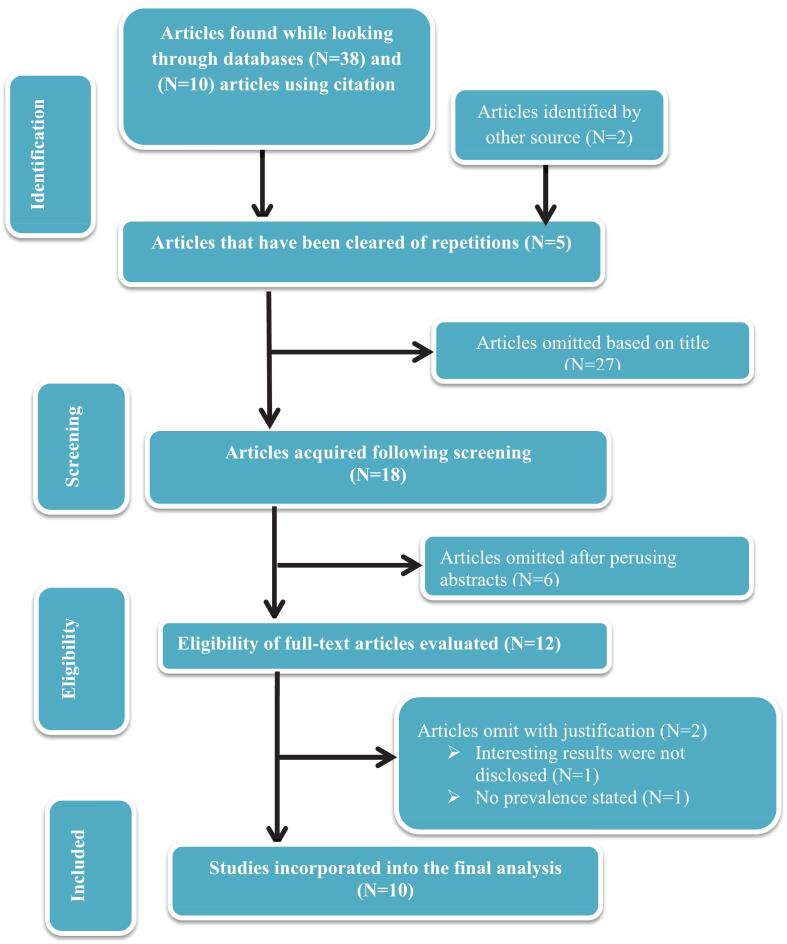
Flow diagram showing the process of searching the literature and choosing studies in the review.

The systematic search conducted between April and June 2025 initially identified 38 studies. An additional 12 studies were identified through the snowballing method, bringing the total to 50 studies for evaluation. After screening titles and abstracts, 18 studies were shortlisted for full-text assessment. Of these, 6 studies were excluded based on abstract review, and a further 2 studies were excluded after full-text review for not meeting the inclusion criteria (see Fig. 1). Ultimately, 10 studies were included in the final analysis, comprising 7 cohort and 3 cross-sectional studies. The selection process was independently conducted by two reviewers (GWG and TTA), who reached full consensus on the included studies.

### 3.2 Characteristics of the studies included in the analysis

Detailed characteristics of the included studies are presented in Table 1. A total of 2798 participants were investigated across the 10 studies. All included studies were observational in design, comprising 7 cohort studies (70%) and 3 cross-sectional studies (30%). The studies were conducted in diverse countries in Africa and were published between 2008 and 2025. Sample sizes varied considerably, ranging from 39 to 1921 participants. Of the 10 studies, 2 focused on adult populations, while the remaining 8 were conducted in pediatric populations.

**Table 1:** Characteristics of primary article describing treatment outcome and its predictors among patients with status epilepticus in Africa (*n* = 10).

Author	Patient age (year)	Years of publications	Country	Study design	Sample size	Treatment used	Prevalence of Death %	Cause of SE
Idro et al.^37^	0–13	2008	Kenya	RC	98	NM	3.1	Infection, malaria, pyogenic meningitis, respiratory tract infections and gastroenteritis
Sadarangani et al.^21^	0.083–13	2008	Kenya	PC	155	NM	15	Infection
Prins et al.^31^	2–9	2014	Kenya	PC	155	All patients on I·V diazepam, and 10.9% of the patients had phenobarbital, carbamazepine, or phenytoin.	7.6	Fever and generalized tonic-clonic seizure type
Sourbron et al.^34^	0.33–10.33	2021	Mozambique	RC	39	91.7% of patients had diazepam or midazolam, 8.3% of patients had phenytoin. Around 35.9% of patient had on second line ASM.	0	CNS infection
Olubosede et al.^38^	0.333–8	2017	Nigeria	CS	39	NM	23.1	Meningitis, fever, malaria, and head injury
Owolabi et al.^32^	18 and above	2014	Nigeria	PC	76	All patients on I·V diazepam, 67% and 56% of the patients had on phenytoin and other ASMs, respectively.	22.4	Non-adherence to ASMs, stroke, and metabolic derangement
Sabo et al.^36^	0.083–14	2025	Nigeria	CS	1921	NM	24.7	Malaria
Shayo et al.^35^	0.083–14	2023	Tanzania	PS	114	78.8% of the patient responded on BZDs, the remainder required both BZDs and phenobarbital	16.7	Meningitis, generalized tonic clonic seizure, and fever
Amare et al.^13^	13 and above	2008	Ethiopia	RC	119	I·V diazepam and phenytoin PO were administered to 95% and 97.5% of patients, respectively, while oral phenobarbital and carbamazepine were given to 24.4% and 11.8%. General anesthesia and mechanical ventilation were required in 10 and 11 patients, respectively.	20.2	CNS infection, and antiseizure medication withdrawal
Abdie et al.^33^	0.083–18	2022	Ethiopia	CS	82	84.1% of patients on I·V diazepam and 15.9% of patients on phenytoin PO as first line. Second line phenobarbital and phenytoin in 3.7% and 73.2% of patients.	9.8	Meningitis, and malaria

ASM—Antiseizure medication, BDZs—Benzodiazepines, NM—Not mentioned, PO—per oral, I·V—Intravenously, CS—Cross-sectional, PC—Prospective cohort, RC—Retrospective cohort.

¥--The initial oral treatment given by nasogastric (NG) tube.

Across the six studies reviewed, intravenous (I·V) diazepam was the most consistently used first-line anti-seizure medication (ASM), administered to the majority of patients in all studies. In studies done in Kenya^31^ and Nigeria,^32^ all patients received I·V diazepam, while in Ethiopia,^33^ 84.1% were treated with I·V diazepam and 15.9% with oral phenytoin. Studies also reported the use of other benzodiazepines, with study done in Mozambique^34^ indicating that 91.7% of patients received either diazepam or midazolam, and study done in Tanzania^35^ showing that 78.8% of patients responded to benzodiazepines alone, while the remainder required additional phenobarbital. Phenytoin, either oral or I·V, was a common second-line agent, with usage ranging from 8.3% in Mozambique^34^ to 97.5% in Ethiopia.^13^ Phenobarbital was used as a second-line agent in several studies, including 10.9% in Kenya,^31^ 24.4% in Ethiopia,^13^ and 3.7% in Ethiopia.^33^ Other ASMs (e.g., carbamazepine) were less frequently used, reported in 11.8% and 56% of patients in Kenya,^31^ Nigeria,^32^ and Ethiopia.^13^ Approximately 35.9% of patients in Mozambique^34^ were managed with second-line ASMs, while a small proportion required intensive care interventions such as general anesthesia and mechanical ventilation.^13^ Overall, the treatment pattern highlights a heavy reliance on benzodiazepines as initial therapy, followed by phenytoin and phenobarbital as second-line options. The mortality rate among individual studies varied from 0^34^ to 24.7%.^36^

In the included studies, infections were the most frequently reported underlying cause of status epilepticus (SE), particularly central nervous system (CNS) infections such as meningitis.^13^, ^21^, ^33^, ^34^, ^35^, ^37^, ^38^ Malaria was another common trigger identified in several studies.^33^, ^36^, ^37^, ^38^ Additional contributing factors included fever and generalized tonic-clonic seizures,^31^, ^35^, ^38^ respiratory tract infections and gastroenteritis,^37^ and head injury.^38^ Other less frequently reported causes included non-adherence to antiseizure medications (ASMs), stroke, and metabolic derangements,^32^ as well as antiseizure medication withdrawal.^13^ These findings highlight the multifactorial nature of SE, with infections and malaria being the predominant causes in the reviewed articles (Table 1).

### 3.3 Study quality

All included studies were independently assessed for methodological quality by three authors (YAW, GWG, and TTA) using the Newcastle-Ottawa Scale (NOS), a standard tool for evaluating observational studies in systematic reviews.^39^ The NOS evaluates three domains: selection, comparability, and outcome, with a maximum score of nine stars (4 for selection, 2 for comparability, and 3 for outcome). Cross-sectional and cohort studies were assessed based on relevant criteria within these domains. Studies scoring ≥7 were considered high quality, those scoring 6 were classified as fair risk of bias, and scoring <6 were considered low quality. Discrepancies were resolved through discussion or with a third reviewer. If a consensus could not be reached, the final score was determined by averaging the two reviewers' scores.

To ensure the inclusion of studies with acceptable methodological quality and low risk of bias, only those that achieved a minimum score of six stars on the NOS were included in this review. This threshold ensured that the findings synthesized in the meta-analysis were based on studies with sound design, reliable outcome measures, and appropriate control for confounders.

Among the 10 included studies, 8^13^, ^21^, ^31^, ^32^, ^34^, ^35^, ^36^, ^37^ (80%) scored at least 7 out of 9 on the Newcastle-Ottawa Quality Assessment Scale (NOQAS) and were considered high quality. These comprised 3 retrospective cohort studies, 4 prospective cohort studies, and 1 cross-sectional study. In contrast, two cross-sectional studies^33^, ^38^ scored 6 out of 9, indicating a fair risk of bias. These lower-quality studies were marked by inadequate statistical methods, unclear sampling procedures, and a lack of control for potential confounders in both study design and analysis (Table S4). During the quality assessment process, we also examined whether the included studies reported ethical approval from an institutional review board or ethics committee and whether participant informed consent was obtained when applicable. As this study is a meta-analysis based solely on previously published studies, no additional ethical approval was required.

### 3.4 Pooled prevalence of mortality and neurological sequelae among patients with status epilepticus in Africa

**Fig. 2:**
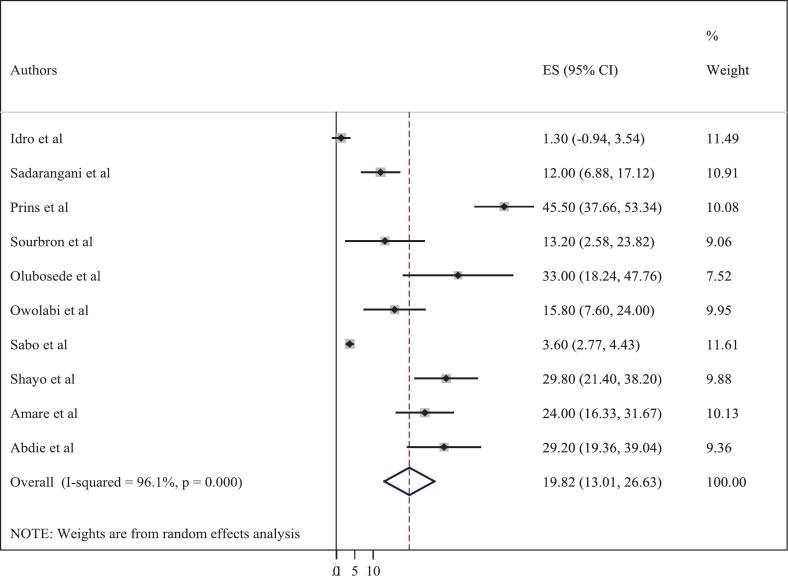
Forest plot showing the pooled prevalence of neurological sequelae among status epilepticus patient in Africa (n = 10).

**Fig. 3:**
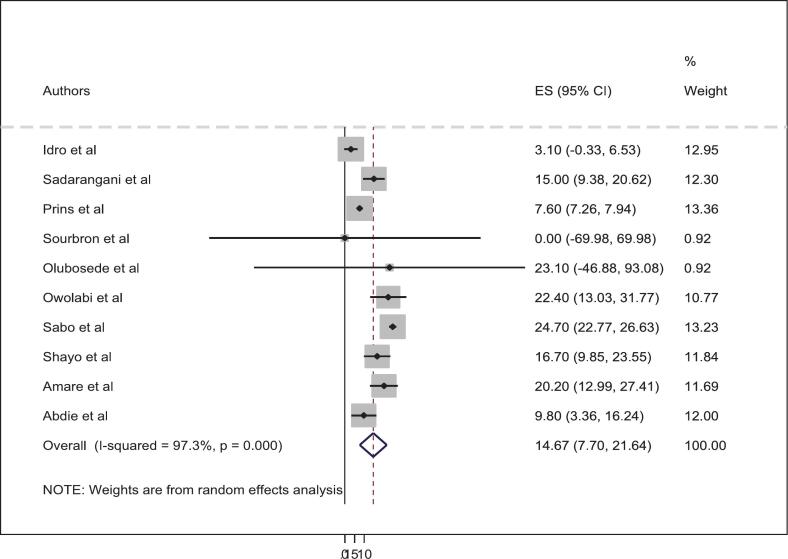
Forest plot showing the pooled prevalence of mortality among status epilepticus patient in Africa (n = 10).

The overall pooled prevalence of neurological sequelae among patients with status epilepticus in Africa was 19.82% (95% CI: 13.01–26.63). However, individual studies conducted in Africa demonstrated substantial variability. The lowest prevalence was reported in a study from Kenya (1.3%),^37^ while the highest, also from Kenya, reached 45.5%^31^ (see Fig. 2). Regarding mortality, the pooled prevalence was 14.67% (95% CI: 7.70–21.64). Both primary and secondary mortality outcomes were combined in this analysis, as event rates were similar across different follow-up periods, which allowed for a comprehensive estimate of SE-related mortality. Similarly, mortality rates varied widely across studies, ranging from 0% in a study conducted in Mozambique^34^ to 24.7% in a study from Nigeria^36^ (see Fig. 3). Overall, this systematic review and meta-analysis included ten studies, which collectively demonstrated substantial heterogeneity (I^2^ = 97.3%, *p* < 0.001).

### 3.5 Publication bias

**Fig. 4:**
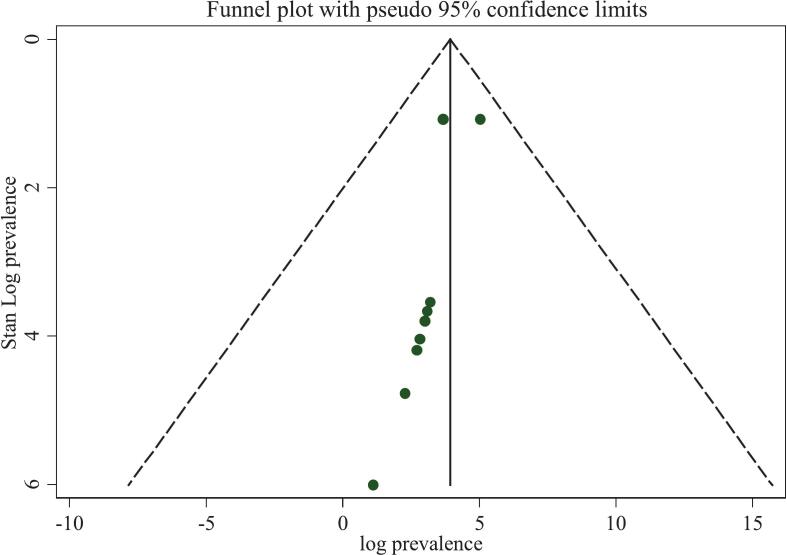
Funnel plot for the publication bias of the included studies for overall all mortality among patients with status epilepticus in Africa (n = 10).

**Fig. 5:**
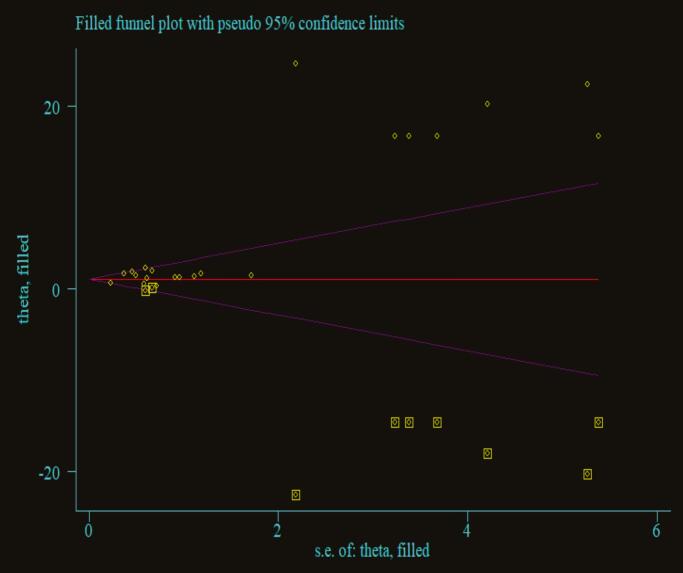
Trim-and-fill funnel plot showing assessment of publication bias among studies reporting mortality in status epilepticus in Africa.

Publication bias was assessed using both visual and statistical methods. The funnel plot appeared slightly asymmetric, with studies clustering predominantly on one side of the mean, suggesting potential small-study effects or possible publication bias (Fig. 4). However, Egger's regression test revealed no statistically significant evidence of publication bias (*p* = 0.052) (Table 2). Given that Egger's test has limited power to detect bias when the number of studies is small (approximately ten), a trim-and-fill analysis was conducted to explore potential missing studies (Fig. 5). The adjusted pooled estimate did not differ substantially from the original, indicating that publication bias was unlikely to have materially influenced the results. To further explore the sources of heterogeneity, both sensitivity and subgroup analyses were performed.

**Table 2:** Egger's test for publication bias in the included studies for overall mortality among patients with status epilepticus in Africa.

Std.Eff.	Coef.	Std.Err.	t	p > |t|	(95%, CI)
Slope	4.634288	0.373319	12.41	0.000	3.773413–5.495164
Bias	−0.4648429	0.2036143	−2.28	0.052	−0.9343784-0.0046926

CI — Confidence interval.

Std. Eff. --- Standardized Effect Size.

Std.Err. — Standard error.

### 3.6 Prevalence of mortality as determined by subgroup analysis

The prevalence of mortality among patients with status epilepticus in Africa varied significantly depending on geographical location, study design, SE definition, and age, according to a subgroup analysis of the primary studies. Based on geographical location, the review showed a higher prevalence in Western Africa (24.61%) compared to Eastern Africa (11.12%), with consistent findings in the former (I^2^ = 0%) and moderate heterogeneity in the latter (I^2^ = 81.4%). By study design, cross-sectional studies reported the highest prevalence (17.8%), followed by prospective cohorts (14.58%) and retrospective cohorts (10.80%), all with high heterogeneity. Regarding age, adults had a higher and more consistent prevalence (21.02%, I^2^ = 0%) than children (12.77%, I^2^ = 97.8%). Subgroup analysis based on the definition of status epilepticus showed that studies using the ≥30-min definition reported a pooled estimate of 11.68 (95% CI: 7.34–16.03; I^2^ = 80.1%), whereas those using the ≥5-min definition reported a higher pooled estimate of 21.40 (95% CI: 13.68–29.12; I^2^ = 79.4%) (Table 3).

**Table 3:** Showing the subgroup analysis per geographical location, study design, and age of the pooled prevalence of mortality among patients with status epilepticus in Africa (2025).

Variable	Sub-group	Number of study	Prevalence (%) (95% CI)	I^2^ (%)	P-value
Geographical location	Eastern Africa	7	11.12(7.04–15.19)	81.4	< 0.0001
	Western Africa	3	24.61(22.72–26.49)	0.0	0.894
Study design	Prospective cohort	4	14.58(7.66–21.50)	86.9	< 0.0001
	Retrospective cohort	3	10.80(−5.02–26.61)	88.7	0.181
	Cross sectional	3	17.8(3.9–31.7)	89.4	0.012
Age	Children	8	12.77(4.81–20.72)	97.8	0.002
	Adult	2	21.02(15.30–26.74)	0.0	0.715
SE definition	>/= 30 min	8	11.68(7.34–16.03)	80.1	< 0.0001
	>/= 5 min	2	21.40(13.68–29.12)	973	< 0.0001

CI—Confidence interval.

I^2^—Chi-square.

The meta-regression analysis revealed that the year of publication is positively associated with the log odds of status epilepticus (log OR coefficient = 0.57, *p* = 0.050). Additionally, a meta regression was conducted to assess the effect of sample size on mortality variation among individual studies. Based on this, the meta-regression analysis shows a significant positive association between sample size and the log odds ratio of status epilepticus. Specifically, as the sample size increases, studies tend to report higher odds of status epilepticus (coefficient = 0.009, *p* = 0.004) (Table 4).

**Table 4:** Meta regression by year of publication and sample size in the included studies among patients with status epilepticus in Africa (2025).

Variables	Coef.	Std. Err.	t	P > |t|	95% CI
Year of publication	0.573	0.280	2.05	0.05	−0.001, 1.15
Cons	−1144.6	563.9	−2.03	0.052	−2301.5, 12.3
Sample size	0.009	0.003	3.11	0.004	0.0031, 0.0150
Cons	7.355	1.792	4.11	0.000	3.679, 11.031

Coef—Coefficient.

Sdt. Err---Standard error.

CI—Confidence interval.

### 3.7 Sensitivity analysis

Sensitivity analysis was performed to explore the sources of heterogeneity and assess the influence of individual studies on the overall pooled prevalence of mortality among children with status epilepticus. The results showed that the exclusion of any single study did not substantially impact the overall estimate, as all values remained within the 95% confidence interval. This indicates that the findings are robust and not unduly influenced by any single study (Table 5). We conducted a sensitivity analysis by excluding the two low-quality studies (Olubosede et al. and Abdie et al.). Following their removal, the pooled mortality estimate increased marginally from 14.67% (95% CI: 7.70–21.64) to 15.26% (95% CI: 7.59–22.93) (Table 6). The level of heterogeneity remained high (I^2^ = 97.9%, *p* < 0.0001), indicating substantial variability among the included studies even after exclusion. The minimal change in the pooled estimate suggests that the overall results are robust and not materially affected by the inclusion of low-quality studies, although considerable heterogeneity persists.

**Table 5:** Sensitivity analysis of mortality in the included studies among patients with status epilepticus in Africa (2025).

Authors	Estimate with 95% CI	Heterogeneity	
		I^2^ (%)	P-value
Idro et al	16.39(8.30, 24.49)	97.5	< 0.0001
Sadarangani et al	14.63(7.00, 22.27)	97.6	< 0.0001
Prins et al.	15.77(7.79, 23.74)	93.7	< 0.0001
Sourbron et al	14.81(7.80, 21.82)	97.6	< 0.0001
Olubosede et al	14.59(7.58, 21.61)	97.6	< 0.0001
Owolabi et al	13.74(6.36, 21.12)	97.5	< 0.0001
Sabo et al	12.38(8.21, 16.54)	80.9	< 0.0001
Shayo et al	14.40(6.87, 21.93)	97.6	< 0.0001
Amare et al	13.94(6.48, 21.40)	97.5	< 0.0001
Abdie et al	15.34(7.72, 22.96)	97.6	< 0.0001

**Table 6:** Sensitivity analysis of pooled mortality estimates after excluding low-quality studies among patients with status epilepticus in Africa (2025).

Authors	Estimate with 95% CI	Heterogeneity	
		I^2^ (%)	P-value
Olubosede et al. and Abdie et al	15.26(7.59–22.93)	97.9	< 0.0001

I^2^ = Chi-square. CI = Confidence interval.

### 3.8 Predictors of mortality among patients with status epilepticus treated with antiseizure medications in Africa

Among the ten studies included in this systematic review and meta-analysis, nine reported both the magnitude and predictors of mortality, while one study focused solely on mortality rates. A wide range of factors were reported to be associated with mortality in patients with status epilepticus, including hypoglycemia, bacterial meningitis, inadequate treatment, non-adherence, seizure type, refractory status epilepticus, seizure duration over 30 min, altered consciousness on admission, fever, hypoxia, electrolyte imbalances (e.g., abnormal potassium), HIV/AIDS and its CNS complications, comorbidities, coma, and acidosis. However, for the purpose of meta-analysis, only variables identified as mortality predictors in at least two original studies were considered. Accordingly, hypoglycemia, bacterial meningitis, and inadequate treatment were included in the quantitative synthesis to evaluate their pooled association with mortality.

**Fig. 6:**
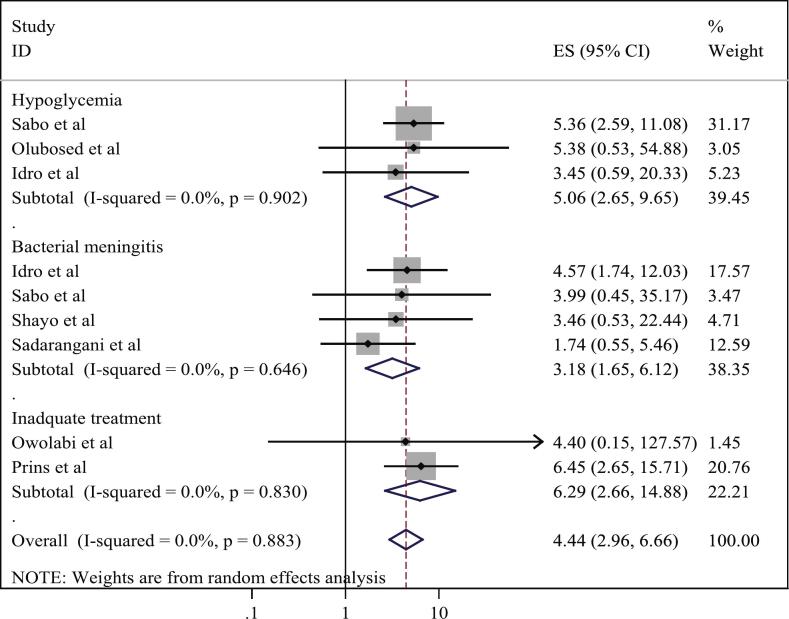
A forest plot showing the combined random-effect size (odds ratio) of various predictors on mortality among patients with status epilepticus in Africa (n = 10).

Three^36^, ^37^, ^38^ studies were included to identify the associations between hypoglycemia and mortality in patients with status epilepticus. Based on the random-effects model, the pooled analysis revealed that patients with hypoglycemia had significantly higher odds of death compared to non-hypoglycemic patients [OR = 5.06; 95% CI: 2.65–9.65]. Similarly, four of these studies^21^, ^35^, ^36^, ^37^ reported a significant relationship between bacterial meningitis and mortality in status epilepticus. The pooled estimate indicated that the odds of death were markedly higher, over threefold, among patients with bacterial meningitis compared to those without [OR = 3.18; 95% CI: 1.65–6.12]. Furthermore, two studies^31^, ^32^ identified inadequate treatment as a significant predictor of mortality. The combined estimate from these studies showed that patients who received inadequate treatment had more than six times the odds of death compared to those who received appropriate treatment [OR = 6.29; 95% CI: 2.66–14.88]. In this review, consistency (no heterogeneity) across studies increases the reliability of the pooled results. Inadequate treatment has the highest pooled effect size, indicating it is the most impactful risk factor among those assessed (Fig. 6). Although all individual studies showed significant results, the pooled meta-analysis of some studies did not. This may be due to variability in effect sizes, high heterogeneity, and the greater weight given to larger studies with less extreme findings. The pooled analysis also accounts for random error and potential publication bias, providing a more conservative and balanced overall estimate.

## 4 Discussion

Status epilepticus (SE) is a common neurological emergency characterized by prolonged seizures, either convulsive or nonconvulsive, which may develop as a complication of epilepsy or occur de novo in nearly half of cases.^40^, ^41^ Despite the adoption of more aggressive treatment protocols, SE remains associated with high mortality, particularly in developing countries, and among adults and patients unresponsive to first-line therapy.^19^ The aim of this review is to determine the pooled prevalence of mortality and its predictors among patients with SE in Africa.

The pooled prevalence of death among patients with SE in Africa was 14.67%, consistent with a systematic review from England reporting 13.0% in all age groups, 15.9% in adults, and 3.6% in children.^19^ A study from Saudi Arabia documented a mortality rate of 18.4%, further supporting our findings.^42^ This reflects the inherently severe nature of SE, which carries a high risk of death regardless of location, and challenges in timely recognition and effective management, particularly in adults. However, the pooled mortality rate in this review was notably higher than that reported in China (1.4%),^43^ the USA^44^ and Germany^45^ (3%). This discrepancy likely reflects differences in healthcare infrastructure, access to advanced neurocritical care, early intervention, continuous EEG monitoring, delayed presentation, limited availability of second- or third-line antiseizure therapies, and a higher prevalence of underlying causes such as CNS infections in African settings.

The pooled prevalence of neurological sequelae among patients with SE in Africa was 19.82%, consistent with a systematic review from Denmark reporting 18.4%.^46^ Patients who developed neurological deficits were primarily diagnosed with cerebral malaria or meningitis, with the most common deficit being regression of previously attained developmental milestones, occurring in nearly half of affected patients. Other reported deficits included cortical blindness, deafness, spasticity, and facial nerve palsy.^38^

Refractory status epilepticus (RSE) was reported in a limited number of the included studies and therefore could not be quantitatively pooled in the present analysis. Nevertheless, available evidence suggests that RSE is an important contributor to poor outcomes. Globally, RSE is strongly associated with increased morbidity and mortality due to prolonged seizure activity, systemic complications, and the need for advanced neurocritical care. In many African settings, limited access to intensive care units, continuous EEG monitoring, and second- or third-line antiseizure medications may further worsen outcomes among patients who develop refractory seizures.^47^, ^48^ Although the available data in this review were insufficient for formal meta-analysis, the findings highlight the need for future studies to systematically report RSE and its outcomes. Improved reporting would allow a more robust assessment of its impact and may inform the development of neurocritical care services and treatment protocols in resource-limited settings.

Subgroup analyses revealed marked differences in mortality across geographic regions, study designs, SE definition, and age groups. The highest mortality was observed in Western Africa (24.61%) and in adult populations (21.01%). Cross-sectional studies reported the highest mortality rates (17.8%) compared to prospective cohort studies (14.58%). These differences highlight the combined effects of demographic, methodological, and health-system factors on SE outcomes, with adults facing higher mortality due to chronic diseases, advanced age, delayed treatment, and more severe underlying conditions.^7^, ^45^, ^49^ Mortality estimates from cross-sectional studies tend to be higher due to selection bias and inclusion of more severe hospital cases.^50^, ^51^ Studies applying the ≥5-min definition of Status Epilepticus are generally expected to report lower mortality due to earlier recognition and prompt treatment, inclusion of less severe cases, and advances in modern management protocols. In contrast, the traditional ≥30-min definition tends to capture more prolonged and severe seizures, which are associated with poorer outcomes. However, our subgroup analysis did not fully support this expectation, as a higher pooled estimate was observed among studies using the ≥5-min definition. This finding should be interpreted with caution, as it may be influenced by the small number of studies in the ≥5-min subgroup, potentially leading to unstable estimates and limiting the reliability of the comparison.

A major methodological feature of this review was the extremely high degree of heterogeneity (I^2^ > 97%) across the included studies. Such substantial heterogeneity warrants careful interpretation and raises questions about the appropriateness of pooling results. The observed variability likely reflects real differences in study populations, definitions of status epilepticus, etiological profiles, diagnostic capacity (particularly access to EEG), treatment protocols,^52^ and levels of healthcare delivery ranging from primary facilities to tertiary referral centers.^53^ Methodological differences between studies may also contribute to the observed variability. Given this level of heterogeneity, the pooled estimate should be interpreted cautiously and viewed primarily as a broad continental average rather than a precise or stable epidemiologic estimate. Therefore, while the pooled findings provide a useful overview of the burden of status epilepticus in Africa, they may mask substantial regional and clinical differences. This underscores the importance of interpreting the results within their local context and highlights the need for more standardized study designs and reporting practices in future research.

Meta-regression analysis suggested a positive association between publication year and the log odds of status epilepticus (log OR coefficient = 0.57, *p* = 0.050). However, this association was only marginally significant, and the 95% confidence interval (−0.001 to 1.15) included zero. Therefore, the finding should be interpreted cautiously and considered exploratory rather than evidence of a true temporal trend. If present, the observed pattern may reflect improved diagnostic practices, increased awareness, or differences in study populations and methodologies over time. Similarly, meta-regression showed a significant positive association between sample size and the log odds ratio of status epilepticus (coefficient = 0.009, *p* = 0.004). However, this should not be interpreted as evidence that larger studies detect a true higher risk. The finding may reflect methodological factors such as referral bias in large tertiary centers, better documentation and diagnostic accuracy, or ecological confounding. Therefore, this association should be interpreted cautiously and considered hypothesis-generating rather than definitive.

Given the observed differences in mortality between adults and children, the clinical pathways leading to SE should be interpreted within age-specific contexts. In adults, SE is more frequently associated with structural brain lesions such as stroke, traumatic brain injury, brain tumors, alcohol withdrawal, and chronic comorbidities including hypertension, diabetes, and renal disease. These conditions often reflect underlying cerebrovascular pathology and systemic instability, which may partly explain the higher mortality observed in adult populations.^48^, ^54^ In contrast, pediatric SE in many African settings is more commonly precipitated by acute infections (e.g., bacterial meningitis, cerebral malaria, and other central nervous system infections), febrile illnesses, and metabolic disturbances such as hypoglycemia. While children may have greater neuroplasticity and potentially better recovery potential, delayed treatment in infection-related SE can still result in significant neurological sequelae. These distinctions underscore the need for age-specific prevention and management strategies—such as strengthened stroke prevention and chronic disease control programs for adults, and improved infection prevention, vaccination coverage, early fever management, and rapid metabolic correction for children.^37^, ^55^, ^56^

The second aim of our study was to identify the factors that contribute to mortality among patients with SE in Africa. The meta-analysis showed that mortality in patients with SE was significantly associated with hypoglycemia and bacterial meningitis. The pooled analysis indicated that patients with hypoglycemia were 5.06 times more likely to experience mortality from status epilepticus compared to those without hypoglycemia. From a pathophysiological perspective, glucose is the primary energy substrate for neuronal metabolism, and hypoglycemia leads to rapid depletion of cerebral energy reserves. During prolonged seizures, cerebral glucose utilization is markedly increased, and the coexistence of hypoglycemia exacerbates neuronal energy failure, leading to excitotoxic injury, mitochondrial dysfunction, and irreversible neuronal damage. Hypoglycemia may also lower the seizure threshold, prolong seizure duration, and impair the effectiveness of antiseizure medications. In children, who have limited glycogen reserves, this effect is particularly pronounced and has been associated with both increased mortality and long-term neurological sequelae.^57^ In African settings, hypoglycemia often occurs in the context of malaria, sepsis, malnutrition, or delayed hospital presentation, underscoring the importance of routine glucose monitoring and prompts correction as a core component of SE management.^58^, ^59^, ^60^ This is in line with previous studies done in United States,^61^ which reported that hypoglycemia is an independent predictor of death in patients with SE. Hypoglycemia was also found to increase the risk of neurological sequelae in a prospective study involving children.^21^ It may also serve as a potential trigger for SE.^12^, ^32^, ^62^ Compared with patients without bacterial meningitis, children with bacterial meningitis are 3.18 times more likely to die, possibly due to the severe inflammation, increased intracranial pressure, and rapid disease progression associated with the condition. In African settings, delayed diagnosis of meningitis is common due to limited access to lumbar puncture, lack of laboratory diagnostic capacity, and late presentation to healthcare facilities. Additionally, the burden of severe CNS infections remains disproportionately high, particularly among children, with frequent overlap between meningitis, encephalitis, and cerebral malaria. Delays in initiating appropriate antimicrobial therapy further compound neurological injury and contribute to poor outcomes.^63^, ^64^ These factors explain why meningitis consistently emerges as both a precipitating cause of SE and an independent predictor of mortality in African and global pediatric populations. This finding is consistent with previous studies in the United States^61^ and Saudi Arabia,^42^ which have identified sepsis as a significant risk factor for mortality in pediatric populations, which may develop secondary to pneumonia or other infectious sources. Moreover, a prospective pediatric study identified meningitis and encephalitis as leading causes of SE, with meningitis also emerging as an independent risk factor for death.^65^ Similar associations have been reported in both prospective^21^ and retrospective^66^ studies. However, cardiovascular disorders are the most common etiology of SE in older individuals (≥ 60 years).^67^

Inadequate treatment of status epilepticus (SE) was associated with a 6.29-fold increase in mortality compared with timely and appropriate management. However, this estimate should be interpreted cautiously because only two studies contributed to the pooled analysis. Although statistical heterogeneity was low (I^2^ = 0%), this may reflect limited statistical power rather than true homogeneity across studies. Across the included studies, the definition of “inadequate treatment” varied, but common elements consistently emerged. These included delayed administration of first-line benzodiazepines, sub-therapeutic dosing, use of suboptimal routes of administration due to limited intravenous access, failure to escalate to second- or third-line antiseizure medications when seizures persisted, and premature discontinuation of therapy due to drug shortages or financial constraints. By synthesizing these elements, it becomes clear that treatment inadequacy reflects cumulative delays and gaps in protocol adherence rather than a single misstep. In many African settings, this inadequacy often arises from delays in administering first-line benzodiazepines, incorrect dosing, or reliance on suboptimal routes of administration due to limited intravenous access. The problem is compounded by the scarcity of second-line antiseizure medications (such as phenytoin, phenobarbital, or valproate), absence of third-line agents, and the early discontinuation of therapy caused by drug shortages or cost constraints. As a result, seizures may persist or become refractory, leading to progressive neuronal injury, pharmaco-resistance, systemic complications, and increased mortality.^52^, ^68^, ^69^ Logistical barriers further exacerbate treatment delays. These challenges align with findings from other studies showing that inadequate treatment—often driven by delays in transport and logistical constraints in emergency units—is strongly associated with higher mortality.^31^, ^32^ This treatment gap may be partly attributed to the high cost of antiseizure medications (ASMs). Socio-cultural factors also play a role. The high cost of antiseizure medications can limit access, and parental beliefs—such as the perception that seizures are caused by spiritual forces and are therefore not responsive to medical treatment—may delay or prevent appropriate care. To reduce seizure duration and associated complications, interventions should educate parents that SE is a treatable medical condition. Furthermore, fostering collaboration between healthcare providers and traditional healers may help improve treatment uptake and strengthen care programs for affected children.70., 71 To better contextualize these findings, the predictors of mortality and neurological sequelae in patients with SE in Africa can be understood within an integrated conceptual framework that links patient-level factors, disease-related mechanisms, and health system-level determinants to clinical outcomes. At the patient level, acute metabolic derangements such as hypoglycemia and underlying etiologies such as bacterial meningitis, cerebral malaria, and other CNS infections directly exacerbate neuronal injury. Hypoglycemia accelerates cerebral energy failure during prolonged seizures, increasing excitotoxic damage and seizure refractoriness, while severe infections contribute to systemic instability, neuroinflammation, raised intracranial pressure, and rapid neurological deterioration. These biological mechanisms increase both immediate mortality risk and the likelihood of long-term neurological sequelae. Importantly, mortality risk must also be understood within the context of pre-hospital care limitations. Delayed caregiver recognition of SE, stigma, reliance on traditional healers, and limited emergency transport systems frequently prolong seizure duration before medical evaluation. Restricted access to and underuse of pre-hospital benzodiazepines further delay seizure termination. Together, these factors substantially contribute to preventable mortality. Therefore, reducing the burden of SE in Africa requires a multi-level approach that extends beyond tertiary hospital management to include community education, improved referral networks, strengthened emergency transport systems, and expanded access to early seizure treatment at primary and pre-hospital levels. Given the limited number of contributing studies and variability in definitions, these findings should be considered preliminary. Future studies should adopt standardized operational definitions of treatment adequacy and systematically report treatment timelines and medication use to allow more robust comparisons across settings.

Globally, the management of SE follows established protocols such as those of the Neurocritical Care Society (NCS),^72^, ^73^ European Federation of Neurological Societies (EFNS),^74^ and American Epilepsy Society (AES),^75^ which emphasize early recognition, prompt benzodiazepine use, and timely escalation to second- and third-line antiseizure medications within 5–30 min of seizure onset. Continuous EEG monitoring and intensive care support are also integral components. Integrate early empirical antibiotic and antimeningitis therapy, together with routine glucose monitoring, into SE management algorithms, particularly for children, since bacterial meningitis and hypoglycemia are major contributors to mortality. Strengthening secure and reliable supply chains for essential benzodiazepines (e.g., IV diazepam, IV lorazepam, rectal diazepam, or IM midazolam for prehospital and primary care use) and affordable second-line antiseizure medications (phenytoin, phenobarbitone) is also critical. Furthermore, SE prevention should be incorporated into broader public health initiatives through enhanced malaria control, routine childhood immunization, and proactive management of hypoglycemia, including clear guidelines for glucose screening and ready access to dextrose in emergency settings.

In contrast, this review shows that implementation of these protocols in African settings remains limited due to delayed presentation, inconsistent drug availability,^76^ limited ambulance system,^77^, ^78^ and inadequate neurocritical care facilities.^79^ The lack of national or regional SE management guidelines further contributes to variable and often suboptimal treatment practices. To address these challenges, context-specific SE management pathways should be developed that align with international recommendations while accounting for local resource constraints. Such protocols should emphasize early recognition at the community and primary care levels, standardized treatment escalation, improved referral systems, and culturally appropriate education to reduce delays in care.

### 4.1 Policy and practice implications

From a public health and health-systems perspective, many of the risk factors identified in this review represent potentially modifiable determinants of mortality. For example, hypoglycemia was a strong predictor of poor outcomes, suggesting that routine point-of-care glucose testing and rapid glucose correction could prevent a proportion of deaths in children presenting with status epilepticus (SE). These interventions are relatively low-cost and feasible even in resource-limited settings. Similarly, timely administration of first-line benzodiazepines through emergency seizure management kits, improved availability of glucose testing strips, and targeted training of emergency and primary care nurses could substantially reduce seizure duration and associated complications.^75^ Task-shifting approaches, where trained nurses or community health workers initiate early seizure management, may further improve outcomes in rural or underserved areas where specialist care is limited.

Improving access to essential antiseizure medications is also critical. Drugs such as diazepam, phenobarbital, and phenytoin are included in the World Health Organization Essential Medicines List,^80^ underscoring their importance in emergency seizure management. Strengthening procurement systems, ensuring reliable drug supply chains, and integrating standardized SE management protocols into emergency care pathways could significantly reduce treatment delays and preventable mortality.

At the policy level, African ministries of health should prioritize the development of national or regional SE management protocols adapted to local resource settings, including guidance on early recognition, antiseizure medication use, glucose monitoring, and empirical treatment for central nervous system infections. Training programs for frontline healthcare workers, particularly in district hospitals and emergency units, should emphasize rapid seizure control, escalation of therapy, and management of common comorbidities. In addition, establishing regional research collaborations and surveillance networks could help standardize SE reporting, monitor outcomes, and inform evidence-based policies. Collectively, these strategies have the potential to improve SE outcomes, reduce preventable deaths, and strengthen neurological care systems across the African continent.

### 4.2 Research gaps and future directions

There are significant gaps in SE research in Africa, with most studies being retrospective, hospital-based, and using varying definitions. Urgently needed are prospective, multicenter studies with standardized SE criteria, including EEG-confirmed non-convulsive SE. Operational research should explore pre-hospital delays, timely benzodiazepine use, and emergency care barriers. Randomized or quasi-experimental designs could evaluate context-specific interventions such as simplified treatment protocols, supply chain improvements, and task-shifting strategies. Filling these gaps will strengthen evidence-based guidelines, inform policy, and improve SE outcomes across the continent.

### 4.3 Strengths and limitations

This study is the first to comprehensively analyze mortality and its predictors in status epilepticus patients across Africa, providing valuable region-specific evidence. By including studies from diverse African regions, the findings are broadly generalizable across the continent. The use of rigorous meta-analytic methods enabled quantitative pooling of mortality rates and the identification of key risk factors such as hypoglycemia and bacterial meningitis. Additionally, subgroup analyses offered important insights into how mortality varies by age, geographic location, publication year, sample size, and study design.

However, several important limitations should be considered when interpreting these findings. First, substantial heterogeneity existed across included studies in terms of design, study populations, case definitions of SE, and outcome measurement. Variability in diagnostic criteria and clinical thresholds may have influenced case ascertainment and contributed to differences in reported mortality and neurological sequelae. Second, data were unavailable from several African regions, limiting geographic representativeness and potentially biasing pooled estimates toward countries with stronger research output and publication capacity.

Third, limited reporting on treatment protocols, including timing of antiseizure medication administration, availability of second- and third-line therapies, access to intensive care, and supportive management, restricted our ability to evaluate how treatment adequacy influenced outcomes. This constraint reduces insight into potentially modifiable system-level determinants of mortality. Fourth, although major databases were systematically searched, the inclusion criteria effectively prioritized English-language articles, which may have excluded relevant studies published in French, Portuguese, or other languages common in Africa. Similarly, some non-indexed or unpublished studies may have been missed, potentially affecting the comprehensiveness and representativeness of the review. While inclusion of one gray literature source helped mitigate publication bias, such sources may lack the methodological rigor of peer-reviewed publications, influencing overall evidence quality.

Fifth, the predominance of observational study designs limits causal inference regarding identified mortality predictors, as residual confounding and unmeasured variables cannot be excluded. Sixth, outcome timing varied considerably across studies: mortality estimates primarily reflect acute, in-hospital case fatality rather than long-term survival, and neurological sequelae were assessed at differing follow-up points—from hospital discharge to several months later—capturing a mix of transient and persistent deficits. Therefore, the pooled estimates largely represent short-term outcomes and should not be interpreted as definitive indicators of long-term prognosis.

Finally, this review should be interpreted in light of potential survivor and case-fatality biases. Most included studies were facility-based and therefore inherently excluded patients who died before reaching healthcare facilities. In many rural and resource-limited African settings, delays in transport, limited emergency medical services, and financial barriers may prevent critically ill patients with SE from accessing care. Consequently, reported mortality rates likely underestimate the true population-level case-fatality, and survivor bias may also influence observed associations between predictors and mortality, as the sickest patients who die pre-hospital are not represented in the analyzed cohorts.

### 4.4 Recommendations for future research

Future research on status epilepticus (SE) in Africa should focus on enhancing methodological rigor, comparability, and regional representativeness. This can be achieved by adopting standardized case definitions, ensuring consistent reporting of treatment variables, and clearly defining follow-up intervals. Prospective, multicenter studies with longitudinal follow-up should be conducted, with systematic reporting of key clinical and management variables such as seizure duration prior to treatment, time to first benzodiazepine administration, availability of second- and third-line antiseizure agents, and ICU admission criteria. Including non-English language publications can improve regional representation. Establishing prospective multicenter registries, measuring time-to-treatment metrics, incorporating non-convulsive SE through improved EEG and neurodiagnostic access, and performing stratified analyses by contextual factors such as HIV status, malaria burden, and ICU availability are also recommended. These strategies will enhance causal inference, strengthen generalizability, and provide actionable evidence to guide clinical practice and health policy, ultimately reducing preventable morbidity and mortality across African SE populations.

## 5 Conclusion

This systematic review and meta-analysis emphasize a significant burden of mortality and neurological sequelae associated with status epilepticus (SE) in African populations. Mortality rates were particularly high among adults and patients with hypoglycemia, bacterial meningitis, or inadequate treatment, highlighting that some of the strongest predictors of poor outcomes are potentially modifiable. These findings underscore critical gaps in timely diagnosis, treatment adequacy, and healthcare infrastructure.

Given the considerable heterogeneity across studies and the observational design of the included research, the reported associations should be interpreted as correlational rather than causal. Caution is advised when generalizing results across all African settings.

Future efforts should prioritize standardized reporting of SE outcomes, the development of context-specific clinical pathways, reliable access to essential antiseizure medications, and culturally sensitive educational interventions. Regional collaborative registries and prospective studies are also recommended to improve data quality, enable robust comparisons, and guide evidence-based strategies to reduce preventable morbidity and mortality.

The following are the supplementary data related to this article.

Supplementary Table S1 PRISMA 2020 checklist for status epilepticus (SE). Supplementary Table S1

Supplementary Table S2 Studies search strategy and entery terms. Supplementary Table S2

Supplementary Table S3 Data extraction format. Supplementary Table S3

Supplementary Table S4 Quality measurment tool by using NOS. Supplementary Table S4

## Authors' contribution

All authors made substantial contributions to the conception, design, data collection, analysis, and interpretation of the study. GWG and TTA extracted the data and assessed the quality of the included studies. GWG drafted the initial version of the manuscript, and all authors contributed to revising and refining the final version. Each author reviewed and approved the final manuscript prior to submission and agreed to be accountable for all aspects of the work. All authors contributed equally to this work.

## CRediT authorship contribution statement

**Gebremariam Wulie Geremew:** Writing – review & editing, Writing – original draft, Visualization, Validation, Supervision, Software, Resources, Project administration, Methodology, Investigation, Formal analysis, Data curation, Conceptualization. **Yilkal Abebaw Wassie:** Writing – review & editing, Writing – original draft, Methodology. **Tirsit Ketsela Zeleke:** Writing – review & editing, Writing – original draft. **Rahel Belete Abebe:** Writing – review & editing, Writing – original draft. **Gebresilassie Tadesse:** Writing – review & editing, Writing – original draft. **Setegn Fentahun:** Writing – review & editing, Writing – original draft. **Sisay Sitotaw Anberbr:** Writing – review & editing, Writing – original draft. **Habtamu Semagne Ayele:** Writing – review & editing, Writing – original draft. **Assefa Kebad Mengesha:** Writing – review & editing, Writing – original draft. **Demis Getachew:** Writing – review & editing, Writing – original draft. **Alemante Tafese Beyna:** Writing – review & editing, Writing – original draft. **Minichil Chanie Worku:** Writing – review & editing, Writing – original draft, Formal analysis. **Abaynesh Fentahun Bekalu:** Writing – review & editing, Writing – original draft, Methodology. **Masho Tigabe Tekle:** Writing – review & editing, Writing – original draft. **Tekletsadik Tekleslassie Alemayehu:** Writing – review & editing, Writing – original draft, Methodology, Formal analysis, Data curation.

## Consent to publication

Not applicable.

## Ethical approval and consent to participate

Not applicable. This study is a systematic review, and no new data were collected directly from human participants. Therefore, ethical approval and informed consent were not required.

## Funding

For this work, the authors did not receive any special funding.

## Declaration of competing interest

None.

## Data Availability

To enhance transparency and reproducibility, all extracted datasets used in this systematic review and meta-analysis, including STATA code for statistical analyses, are available from the corresponding author upon reasonable request. Supplementary tables (Table S3) contain the full extracted raw data for each included study. A PRISMA flowchart (Fig. 1) summarizes the study selection process, with explicit numbers of records identified, duplicates removed, screened, assessed for eligibility, and included in the final analysis.
