# Supplementary material for: Treatment outcome and its predictors among patients with status epilepticus in Africa: A systematic review and meta-analysis
Source: Explor Res Clin Soc Pharm. 2026 Apr 2;22:100738. doi: 10.1016/j.rcsop.2026.100738 (PMC13091730; doi:10.1016/j.rcsop.2026.100738)
Supplement: Supplementary Table S1 — PRISMA 2020 checklist for status epilepticus (SE). [file mmc1.docx]

| **Section and Topic** | | **Item #** | **Checklist item** | **Location where item is reported** |
| --- | --- | --- | --- | --- |
| **TITLE** | | | |  |
| Title | | 1 | **Treatment outcome and its predictors among patients with status epilepticus in Africa: a systematic review and meta-analysis** | 1 |
| **ABSTRACT** | | | |  |
| Abstract | | 2 | **Abstract**  **Background:** Status epilepticus (SE) is a life-threatening emergency requiring urgent care to prevent serious complications or death. Despite treatment advances, mortality remains high in Africa due to multiple risk factors. This review aims to assess treatment outcomes and their predictors among SE patients in Africa.  **Methods:** A comprehensive literature search was conducted using the following databases: African Journals Online, Hinari, Google Scholar, PubMed, Science Direct, EMBASE, Cochrane Database, Sci-Hub, and Scopus. The quality of the included studies was assessed using the Newcastle–Ottawa Scale (NOS) checklist. Data on study characteristics and prevalence estimates were pooled using a random-effect meta-analysis, with additional subgroup and sensitivity analyses performed. Potential publication bias was evaluated through both visual inspection and statistical methods.  **Results:** This review included 10 studies and found that the overall prevalence of mortality and neurological sequelae among patients with SE in Africa was 14.67% (95% CI: 7.70–21.64) and 19.82% (95% CI: 13.01–26.63), respectively. However, considerable heterogeneity was observed, influenced by factors such as geographic region, study design, sample size, and patient age. Subgroup analyses indicated that the highest mortality prevalence was reported in Western Africa at 24.61% (95% CI: 22.72–26.49), in cross-sectional studies at 17.8% (95% CI: 3.9–31.7), and among adult populations at 21.02% (95% CI: 15.30–26.74). Meta-regression analysis revealed a positive association between sample size and the log odds of SE (coefficient = 0.009, p = 0.004). Furthermore, mortality was significantly associated with hypoglycemia [OR = 5.06 (95% CI: 2.65–9.65)], bacterial meningitis [OR = 3.18 (95% CI: 1.65–6.12)], and inadequate treatment [OR = 6.29 (95% CI: 2.66–14.88)].  **Conclusion:** This review reveals a substantial burden of mortality and neurological sequelae associated with status epilepticus, especially in Western Africa, and adult population. Hypoglycemia, bacterial meningitis, and inadequate treatment were independent predictors of mortality in patients with status epilepticus. | 2 |
| **INTRODUCTION** | | | |  |
| Rationale | | 3 | Africa faces a range of unique healthcare challenges that significantly impact the management of status epilepticus (SE), including limited access to diagnostic tools, delays in treatment initiation, shortages of antiepileptic medications, and a critical scarcity of neurologists. These systemic barriers may adversely affect treatment outcomes and are likely to influence the predictors of morbidity and mortality associated with SE in the region. Despite the increasing burden of epilepsy and SE in Africa, there is a lack of comprehensive, region-specific data to inform clinical practice and health policy. As a result, clinicians and decision-makers are often forced to rely on evidence derived from high-income settings, which may not be applicable to African healthcare contexts. This review seeks to address this critical gap by synthesizing the available evidence on treatment outcomes and their predictors among patients with SE across Africa. By consolidating findings from existing studies, the review will offer valuable insights into the regional burden, mortality rates, and key factors influencing treatment outcomes. These findings will aid healthcare providers in identifying high-risk patients, refining clinical decision-making, and optimizing management strategies. Moreover, the study will support health policymakers and stakeholders by highlighting gaps in care and guiding the development of context-specific, evidence-based protocols. Ultimately, this research aims to enhance patient outcomes, reduce SE-related complications, and strengthen neurological care systems throughout the continent. | 4, 5 |
| Objectives | | 4 | The purpose of this review is to systematically and quantitatively summarize the pooled prevalence of mortality and its predictors among patients with SE in Africa. | 4, 5 |
| **METHODS** | | | |  |
| Eligibility criteria | | 5 | All included studies were conducted in African settings, and only facility-based observational study designs were considered. The study populations comprised both children and adults. Both published and unpublished articles that reported treatment outcomes and their associated determinants among children with status epilepticus were eligible for inclusion. Moreover, gray literature was incorporated to minimize publication bias. Although the database searches were limited to articles published in English, no language restrictions were applied during the selection process.  The exclusion criteria encompassed studies lacking clearly defined primary outcomes, as well as systematic reviews, meta-analyses, editorials, and commentaries. This strategy ensured the inclusion of high-quality, peer-reviewed research that directly informed the understanding of treatment outcomes and risk factors associated with status epilepticus in the target population. | 6 |
| Information sources | | 6 | We looked for free publications using databases such as PubMed, Science Direct, EMBASE, Cochrane Database, Sci-Hub, Scopus, Hinari, and Google Scholar. As well as by using snowball techniques. | 6 |
| Search strategy | | 7 | The MESH term used for the search was ((treatment outcome) OR (Outcome) OR (Prognosis) OR (In hospital mortality) OR (Death) AND (Determinants) OR (Predictors) OR (Associated factors) OR (Factors) AND (Status epilepticus) OR SE AND (Africa) | 6, 7 |
| Selection process | | 8 | Using the PRISMA standard, the two authors (GWG and TTA) independently assessed whether primary studies were eligible to be included in this review.  Two data extractors (GWG and TTA) used a standardized data extraction checklist using Microsoft Excel to extract the data. The author name, publication year, nation, study design, population, seizure stratifications, sample size, and outcome measurement were all included in the data extraction checklist for the first outcome (magnitude). The log OR for each factor was computed based on the results of the initial study after data were retrieved in the format of two by two tables for the second outcome (associated factors). By involving a third and fourth reviewer (TKZ, and YAW), disagreements between two independent reviewers were settled. The entire process of data extraction and synthesis has been supervised by GT and SF. | 7 |
| Data collection process | | 9 | Two data extractors (GWG and TTA) used a standardized data extraction checklist using Microsoft Excel to extract the data. The author name, publication year, nation, study design, population, seizure stratifications, sample size, and outcome measurement were all included in the data extraction checklist for the first outcome (magnitude). The log OR for each factor was computed based on the results of the initial study after data were retrieved in the format of two by two tables for the second outcome (associated factors). By involving a third and fourth reviewer (TKZ, and YAW), disagreements between two independent reviewers were settled. The entire process of data extraction and synthesis has been supervised by GT and SF. | 7 |
| Data items | | 10a | The results of this meta-analysis and systematic review provide insight into the prevalence of mortality and its associated factors among patients with SE. Mortality was measured through direct reporting in the source studies. Additionally, we examined mortality in a separate study where participants receiving outpatient treatment for seizure had their mortality documented as a potential factor for other outcomes. In the original study, mortality was assessed through scheduled visits to either an inpatient or outpatient clinic. Articles published up until Jul 30, 2025 were included. | 6, 8 |
|  |  | 10b | Observational study design was included. Factors which affect mortality in patients with SE were also extracted from each study. | 6, 7 |
| Study risk of bias assessment | | 11 | The quality evaluation tool to be used is the Newcastle Ottawa Quality Assessment Scale (NOQAS) (26) for cohort studies and its modified version that assesses cross-sectional studies (27). The NOQAS for cohort studies consists of three main parts: ascertaining exposure (3 items), selecting the study groups (4 items), and determining group comparability (2 items). This scale has a maximum potential score of nine stars if each item is given one star. The Modified Newcastle Ottawa Scale (MNOQS) has three primary components: determining the outcome of interest [(2 items), with 3 score], selecting the study groups [(4 items), 4 score], and comparing the groups [(2 items), 2 score]. The highest potential score on this scale for a single study is 9 **(S4 Table)**. The quality of the included studies was independently evaluated and rated by two authors (GWG and TTA). A study was considered high-quality if it scored ≥ 7 on the MNOQS and was deemed to have a low risk of bias. Any disagreements between the authors will be resolved through discussion. The type of bias will be identified for each study. | 8 |
| Effect measures | | 12 | The data extracted from the Microsoft Excel 2019 format was imported into STATA version 14 for analysis. To assess the variability of primary research, the I^2^ test was utilized. The chi-square values of 0, 25, 50, and 75% indicated no, low, moderate, and high heterogeneity, respectively. Data were pooled and a random effect meta-analysis model was applied to estimate the pooled prevalence and its predictors among patients with SE, and the result was presented using forest plot and odds ratio(OR) with 95% confident interval(CI). | 7, 9 |
| Synthesis methods | | 13a | During our initial search, we identified 38 studies in electronic databases. Using the snowball approach, we uncovered an additional 12 studies that were missed by the initial search, bringing the total to 50 studies for evaluation.  After screening by titles, 18 studies were selected for further review. Upon closer inspection, 6 were excluded after evaluating their full abstracts. We then assessed the remaining 12 studies in full text and excluded 2 more that did not meet our inclusion criteria **(see Fig. 1)**. The two reviewers, GWG and TTA, reached unanimous agreement to include the remaining 10 studies (7 cohort and 3 cross-sectional) in the final analysis. | 10 |
|  |  | 13b | NA |  |
|  |  | 13c | NA |  |
|  |  | 13d | Heterogeneity between studies was assessed by computing chi-square (I^2^) test statistics. The I^2^ values of 0, 25, 50, and 75% were considered as no, low, moderate, and high heterogeneities, respectively. | 14 |
|  |  | 13e | To assess the variability of primary research, the I^2^ test was utilized. The chi-square values of 0, 25, 50, and 75% indicated no, low, moderate, and high heterogeneity, respectively | 9 |
|  |  | 13f | A sensitivity analysis was conducted to assess robustness of the synthesized results. | 19 |
| Reporting bias assessment | | 14 | Begg’s funnel plot and Egger's regression were used to check publication bias and a p-value less than <0.05 was considered as statically significant. | 14-18 |
| Certainty assessment | | 15 | NA |  |
| **RESULTS** | | | |  |
| Study selection | 16a | | During our initial search, we identified 38 studies in electronic databases. Using the snowball approach, we uncovered an additional 12 studies that were missed by the initial search, bringing the total to 50 studies for evaluation.  After screening by titles, 18 studies were selected for further review. Upon closer inspection, 6 were excluded after evaluating their full abstracts. We then assessed the remaining 12 studies in full text and excluded 2 more that did not meet our inclusion criteria **(see Fig. 1)**. The two reviewers, GWG and TTA, reached unanimous agreement to include the remaining 10 studies (7 cohort and 3 cross-sectional) in the final analysis. | 10 |
|  | 16b | | Full version of articles reading was not possible in this case the article becomes excluded. |  |
| Study characteristics | 17 | | Detailed characteristics of the included studies are presented in **Table 1**. A total of 2,798 participants were investigated across the 10 studies. All included studies were observational in design, comprising 7 cohort studies (70%) and 3 cross-sectional studies (30%). The studies were conducted in diverse countries in Africa and were published between 2008 and 2025. Sample sizes varied considerably, ranging from 39 to 1,921 participants. Of the 10 studies, 2 focused on adult populations, while the remaining 8 were conducted in pediatric populations.  Across the six studies reviewed, intravenous (I.V) diazepam was the most consistently used first-line anti-seizure medication (ASM), administered to the majority of patients in all studies. In study done in Kenya (34) and Nigeria (35), all patients received I.V diazepam, while in Ethiopia (36), 84.1% were treated with I.V diazepam and 15.9% with oral phenytoin. Studies also reported the use of other benzodiazepines, with study done in Mozambique (37) indicating that 91.7% of patients received either diazepam or midazolam, and study done in Tanzania (38) showing that 78.8% of patients responded to benzodiazepines alone, while the remainder required additional phenobarbital. Phenytoin, either oral or I.V, was a common second-line agent, with usage ranging from 8.3% in Mozambique (37) to 97.5% in Ethiopia (13). Phenobarbital was used as a second-line agent in several studies, including 10.9% in Kenya (34), 24.4% in Ethiopia (13), and 3.7% in Ethiopia (36). Other ASMs (e.g., carbamazepine) were less frequently used, reported in 11.8% and 56% of patients in Kenya (34), Nigeria (35), and Ethiopia (13). Approximately 35.9% of patients in Mozambique (37) were managed with second-line ASMs, while a small proportion required intensive care interventions such as general anesthesia and mechanical ventilation (13). Overall, the treatment pattern highlights a heavy reliance on benzodiazepines as initial therapy, followed by phenytoin and phenobarbital as second-line options. The mortality rate among individual studies varied from 0 (37) to 24.7% (38).  In the included studies, infections were the most frequently reported underlying cause of status epilepticus (SE), particularly central nervous system (CNS) infections such as meningitis (13, 21, 36-40). Malaria was another common trigger identified in several studies (36, 39-41). Additional contributing factors included fever and generalized tonic-clonic seizures (34, 38, 40). Respiratory tract infections and gastroenteritis (39), and head injury (40). Other less frequently reported causes included non-adherence to antiseizure medications (ASMs), stroke, and metabolic derangements (35), as well as antiseizure medication withdrawal (13). These findings highlight the multifactorial nature of SE, with infections and malaria being the predominant causes in the reviewed articles (**Table 1**). | 10 |
| Risk of bias in studies | 18 | | All included studies were independently assessed for methodological quality by three authors (YAW, GWG, and TTA) using the Newcastle-Ottawa Scale (NOS), a standard tool for evaluating observational studies in systematic reviews (42). The NOS evaluates three domains: selection, comparability, and outcome, with a maximum score of nine stars (4 for selection, 2 for comparability, and 3 for outcome). Cross-sectional and cohort studies were assessed based on relevant criteria within these domains. Studies scoring ≥7 were considered high quality, those scoring 6 were classified as fair risk of bias, and scoring < 6 were considered as low quality. Discrepancies were resolved through discussion or with a third reviewer.  Among the 10 included studies, 8 (13, 21, 34, 35, 37-39, 41) (80%) scored at least 7 out of 9 on the Newcastle-Ottawa Quality Assessment Scale (NOQAS) and were considered high quality. These comprised 3 retrospective cohort studies, 4 prospective cohort studies, and 1 cross-sectional study. In contrast, two cross-sectional studies (36, 40) scored 6 out of 9, indicating a fair risk of bias. These lower-quality studies were marked by inadequate statistical methods, unclear sampling procedures, and a lack of control for potential confounders in both study design and analysis **(Table S4)**. | 7-8 |
| Results of individual studies | 19 | | \| Author \| Years of publications \| Country \| Study design \| Sample size \| Treatment used \| Prevalence of Death % \| Cause of status epilepticus \| \| --- \| --- \| --- \| --- \| --- \| --- \| --- \| --- \| \| Idro et al (39) \| 2008 \| Kenya \| RC \| 98 \| NM \| 3.1 \| Infection, malaria, pyogenic meningitis, respiratory tract infections and gastroenteritis \| \| Sadarangani et al (21) \| 2008 \| Kenya \| PC \| 155 \| NM \| 15 \| Infection \| \| Prins et al (34) \| 2014 \| Kenya \| PC \| 155 \| All patients on I.V diazepam, and 10.9% of the patients had phenobarbital, carbamazepine, or phenytoin. \| 7.6 \| Fever and generalized tonic-clonic seizure type \| \| Sourbron et al (37) \| 2021 \| Mozambique \| RC \| 39 \| 91.7% of patients had diazepam or midazolam, 8.3% of patients had phenytoin. Around 35.9% of patient had on second line ASM. \| 0 \| CNS infection \| \| Olubosede et al (40) \| 2017 \| Nigeria \| CS \| 39 \| NM \| 23.1 \| Meningitis, fever, malaria, and head injury \| \| Owolabi et al (35) \| 2014 \| Nigeria \| PC \| 76 \| All patients on I.V diazepam, 67% and 56% of the patients had on phenytoin and other ASMs, respectively. \| 22.4 \| Non-adherence to ASMs, stroke, and metabolic derangement \| \| Sabo et al (41) \| 2025 \| Nigeria \| CS \| 1921 \| NM \| 24.7 \| Malaria \| \| Shayo et al (38) \| 2023 \| Tanzania \| PS \| 114 \| 78.8% of the patient responded on BZDs, the remainder required both BZDs and phenobarbital \| 16.7 \| Meningitis, generalized tonic clonic seizure, and fever \| \| Amare et al (13) \| 2008 \| Ethiopia \| RC \| 119 \| I.V diazepam and phenytoin PO were administered to 95% and 97.5% of patients, respectively, while oral phenobarbital and carbamazepine were given to 24.4% and 11.8%. General anesthesia and mechanical ventilation were required in 10 and 11 patients, respectively. \| 20.2 \| CNS infection, and antiseizure medication withdrawal \| \| Abdie et al (36) \| 2022 \| Ethiopia \| CS \| 82 \| 84.1% of patients on I.V diazepam and 15.9% of patients on phenytoin PO as first line. Second line phenobarbital and phenytoin in 3.7% and 73.2% of patients. \| 9.8 \| Meningitis, and malaria \| | 12 |
| Results of syntheses | 20a | | During our initial search, we identified 38 studies in electronic databases. Using the snowball approach, we uncovered an additional 12 studies that were missed by the initial search, bringing the total to 50 studies for evaluation.  After screening by titles, 18 studies were selected for further review. Upon closer inspection, 6 were excluded after evaluating their full abstracts. We then assessed the remaining 12 studies in full text and excluded 2 more that did not meet our inclusion criteria **(see Fig. 1)**. The two reviewers, GWG and TTA, reached unanimous agreement to include the remaining 10 studies (7 cohort and 3 cross-sectional) in the final analysis. | 10 |
|  | 20b | | The test statistics (I-squared = 97.3%, P-value < 0.0001) indicate that all 10 studies included in the current systematic review and meta-analysis exhibit significant levels of heterogeneity **(Figure 2).** To assess the presence of publication bias in the included studies, two methods were utilized. Firstly, a funnel plot **(Figure 3)** was created to demonstrate the symmetrical distribution and lack of publication bias in the included studies. Additionally, Egger's test was conducted to further evaluate the presence of publication bias, with a resulting p-value of 0.052 **(Table 2)**. To better understand the sources of heterogeneity, subgroup analysis and sensitivity analysis were performed. | 12-14 |
|  | 20c | | Possible cause of heterogeneity in this review was study design, age, and geographic location.  The prevalence of mortality among patients with status epilepticus in Africa varied significantly depending on the geographical location, study design, and age, according to a subgroup analysis of the primary studies. Based on geographical location, the review showed that higher prevalence in Western Africa (24.61%) compared to Eastern Africa (11.12%), with consistent findings in the former (I² = 0%) and moderate heterogeneity in the latter (I² = 81.4%). By study design, cross-sectional studies reported the highest prevalence (17.8%), followed by prospective cohorts (14.58%) and retrospective cohorts (10.80%), all with high heterogeneity. Regarding age, adults had a higher and more consistent prevalence (21.02%, I² = 0%) than children (12.77%, I² = 97.8%) (**Table 3**). The majority of the included studies (60%) defined status epilepticus (SE) based on the International League against Epilepsy (ILAE) criteria, describing it as a seizure lasting 30 minutes or longer. In contrast, two studies (37, 41) defined SE as a generalized tonic-clonic seizure or focal to bilateral tonic-clonic seizure lasting more than 5 minutes, or as a cluster of seizures without recovery to baseline between episodes. Two additional studies (38, 39) did not provide a clear definition of SE. | 14-17 |
|  | 20d | | By carefully removing one author or one study from the analysis, sensitivity analysis was used in the current systematic review and meta-analysis to investigate the heterogeneity of those studies and determine the effect of each study on the pooled prevalence of mortality among patients with SE. The fact that every value is contained within the expected 95% confidence interval implies that the prevalence of this review was not considerably altered by the removal of one study. | 19 |
| Reporting biases | 21 | | NA |  |
| Certainty of evidence | 22 | | Meta-regression analysis revealed a positive association between sample size and the log odds of SE (coefficient = 0.009, p = 0.004). Furthermore, mortality was significantly associated with hypoglycemia [OR = 5.06 (95% CI: 2.65–9.65)], bacterial meningitis [OR = 3.18 (95% CI: 1.65–6.12)], and inadequate treatment [OR = 6.29 (95% CI: 2.66–14.88)]. | 23-24 |
| **DISCUSSION** | | | |  |
| Discussion | | 23a | Status epilepticus (SE) is a common neurological emergency characterized by prolonged seizures, either convulsive or nonconvulsive, which may develop as a complication of epilepsy or occur de novo in nearly half of cases (43, 44). Despite the adoption of more aggressive treatment protocols, SE remains associated with high mortality, particularly in developing countries, and especially among adults and patients who do not respond to first-line therapy (19). The aim of this review is to determine the pooled prevalence of mortality and its predictors among patients with SE in Africa. Based on this, the findings of this review suggest that the pooled prevalence of death among patients with SE in Africa was 14.67%. This is consistent with another systematic review done in England (19), the mortality of status epilepticus was 13.0% in all age group, 15.9% in adult and 3.6% in children. The possible justification may be attributed to the inherently severe nature of status epilepticus, which carries a high risk of death regardless of geographic location. Additionally, despite disparities in healthcare infrastructure, both settings may face challenges in timely recognition and effective management of SE, especially in adult populations where mortality is notably higher. However, the pooled mortality rate observed in this review was notably higher than that reported in studies from China (1.4%) (45), USA (46) and Germany (47) (3%). This discrepancy may be attributed to differences in healthcare infrastructure, availability of advanced neurocritical care, early intervention, and availability of continuous EEG monitoring, which are more accessible in high-income countries. Moreover, delayed presentation, limited access to second-line or third-line antiepileptic therapies, and higher prevalence of underlying causes such as CNS infections in African settings may further contribute to the increased mortality.  In this review, the pooled prevalence of neurological sequalae among patients with SE in Africa was 19.82%. This result is consistent with a systematic review done in Denmark (48), 18.4% of patients with SE develop neurological sequalae. The patient who developed neurological deficits was primarily diagnosed with cerebral malaria and meningitis. The most common deficit observed was regression of previously attained developmental milestones, occurring in nearly half of the patient. Other reported deficits included cortical blindness, deafness, spasticity, and facial nerve palsy (40).  This review’s subgroup analysis noted extreme differences in reported mortality rates across geographically defined groups, along with study type, and age demographics among patient with SE in Africa. The highest mortality was noted in Western Africa (24.61%) and in studies focusing on adult populations (21.01%). As for study design, the most significant mortality rates were noted in cross-sectional studies (17.8%) and in prospective cohort studies (14.58%). These results provide insight into both geographic and other methodological differences that exist with respect to the mortality level. From a mortality perspective, the higher mortality in adults may indicate a greater burden of mortality due to chronic diseases, advanced age, delayed treatment, and more severe underlying conditions. This is supported by broader evidence indicating that adult SE mortality in low- and middle-income countries often exceeds 20–25%, with adult and elderly patients consistently showing significantly higher mortality rates compared to children (47, 49, 50). Similarly, mortality estimates derived from cross-sectional studies tend to be higher, ranging from 11% to over 40%, likely due to selection bias and the inclusion of more severe or acute hospital cases (51, 52).  The second aim of this systematic review and meta-analysis was to identify the factors that contribute to mortality among SE patient in Africa. The meta-analysis showed that mortality in patient with SE was significantly associated with hypoglycemia and bacterial meningitis. The pooled analysis indicated that patients with hypoglycemia were 5.06 times more likely to experience mortality from status epilepticus compared to those without hypoglycemia. This is in line with previous studies done in United States (53), which reported hypoglycemia is an independent predictor of death in patients with SE. Hypoglycemia was also found to increase the risk of neurological sequelae in a prospective study involving children (21). It may also serve as a potential trigger for SE (12, 35, 54). Compared with patients without bacterial meningitis, children with bacterial meningitis are 3.18 times more likely to die, possibly due to the severe inflammation, increased intracranial pressure, and rapid disease progression associated with the condition. This finding is consistent with previous studies in United States (53), which have identified sepsis as a significant risk factor for mortality in pediatric populations, which may develop secondary to pneumonia or other infectious sources. Moreover, a prospective pediatric study identified meningitis and encephalitis as leading causes of SE, with meningitis also emerging as an independent risk factor for death (55). Similar associations have been reported in both prospective (21) and retrospective (56) studies.  Furthermore, inadequate treatment of status epilepticus was associated with a 6.29-fold increase in the risk of death compared to adequate management. This finding is consistent with other studies, which have shown that inadequate treatment, often resulting from delays in transportation and logistical challenges in emergency medical units, is significantly associated with increased mortality (34, 35). This treatment gap may be partly attributed to the high cost of antiseizure medications (ASMs). Additionally, parental beliefs and perceptions regarding epilepsy, such as the view that seizures are caused by spiritual forces and therefore not responsive to medical treatment, may also contribute to delays or avoidance of appropriate care (57, 58). To reduce seizure episode and their associated complications in SE, interventions should focus on educating parents to recognize as it is a treatable medical condition. Additionally, fostering collaboration between healthcare providers and traditional healers may help improve treatment programs and enhance care for affected children (34).  Overall, this systematic review and meta-analysis reveal an alarming burden of mortality and neurological complications among African patients with status epilepticus. The findings underscore the urgent need for improved early detection, timely access to effective treatment, and targeted public health interventions. Addressing modifiable risk factors such as hypoglycemia, bacterial meningitis, and treatment delays, alongside culturally sensitive community education and strengthening healthcare infrastructure, could substantially reduce SE-related morbidity and mortality across the continent. | 22-24 |
|  |  | 23b | Strength and limitations This review is the first to comprehensively analyze mortality and its predictors in status epilepticus patients across Africa, providing valuable region-specific evidence. By including studies from diverse African regions, the findings are broadly generalizable across the continent. The use of rigorous meta-analytic methods enabled quantitative pooling of mortality rates and the identification of key risk factors such as hypoglycemia and bacterial meningitis. Additionally, subgroup analyses offered important insights into how mortality varies by age, geographic location, publication year, sample size, and study design.  However, this review has some limitations. The included studies varied in design, populations, and definitions of status epilepticus, which contributed to heterogeneity in the results. Data scarcity from certain African regions may limit the representativeness of the findings and introduce bias toward countries with more published research. Furthermore, insufficient detail on treatment protocols restricted the ability to fully assess the impact of treatment adequacy on mortality. Language restrictions may have excluded relevant studies, but there is no language restriction faced during searching, and the predominance of observational study designs limits the ability to draw causal inferences regarding mortality predictors. Although one gray literature source (41) was included, such sources typically lack the rigorous peer-review process of published articles, which may impact the quality of evidence. However, their inclusion can help reduce publication bias and offer a more comprehensive view of the topic.  It is essential to acknowledge these limitations in greater depth, especially regarding their potential influence on the results. Future research should aim to address these gaps by promoting consistent reporting of key variables and including studies published in multiple languages to improve the generalizability of the findings. | 24 |
|  |  | 23c | NA |  |
|  |  | 23d | This systematic review and meta-analysis reveal a substantial burden of mortality and neurological sequelae associated with status epilepticus in Africa. The high mortality rates, especially among adults and those with comorbid hypoglycemia or bacterial meningitis, underscore critical gaps in timely diagnosis, adequate treatment, and healthcare infrastructure. Addressing modifiable risk factors, improving access to antiseizure medications, and integrating culturally sensitive education for families are essential steps toward reducing SE-related morbidity and mortality. | 22 |
| **OTHER INFORMATION** | | | |  |
| Registration and protocol | | 24a | The review was registered with the international prospective register of systematic reviews (PROSPERO) under the registration number “CRD420251102193”. |  |
|  |  | 24b | The systematic review and meta-analysis were conducted and reported following the Preferred Reporting Items for Systematic Reviews and Meta-Analysis (PRISMA) guidelines for observational studies |  |
|  |  | 24c | NA |  |
| Support | | 25 | For this work, the authors did not receive any special funding. | 26 |
| Competing interests | | 26 | The authors declared that there is no conflict of interest in this review. | 26 |
| Availability of data, code and other materials | | 27 | The manuscript and its supporting information files contain all pertinent data. The corresponding author can provide the data that were analyzed and used to construct this work upon reasonable request. | 26 |

*From:*  Page MJ, McKenzie JE, Bossuyt PM, Boutron I, Hoffmann TC, Mulrow CD, et al. The PRISMA 2020 statement: an updated guideline for reporting systematic reviews. BMJ 2021;372:n71. doi: 10.1136/bmj.n71
