# Supplementary material for: Treatment outcome and its predictors among patients with status epilepticus in Africa: A systematic review and meta-analysis
Source: Explor Res Clin Soc Pharm. 2026 Apr 2;22:100738. doi: 10.1016/j.rcsop.2026.100738 (PMC13091730; doi:10.1016/j.rcsop.2026.100738)
Supplement: Supplementary Table S2 — Studies search strategy and entery terms. [file mmc2.docx]

**Table S2: Studies search strategies and entry terms from different electronic databases on treatment outcome and its predictors among patients with Status epilepticus in Africa 2025.**

| **No** | **Search Syntax** |
| --- | --- |
| 1 | (“Treatment outcome” OR (Fatality) OR (Death) OR (Prognosis) OR “Outcomes”) AND (“predictors” OR “factors” OR “associated factors” OR “determinants”) AND (“status epilepticus” OR “SE”) AND (“Africa”) |
| 2 | Incidence AND (Factors OR predictor OR characteristics OR determinant OR component) AND Mortality OR Death OR Fatality OR Outcome OR Treatment outcome AND Status epilepticus OR SE AND Africa. |
| **Additionally, we conducted the following narrowed searches to ensure that no studies were overlooked:** | |
| 3 | Prevalence AND Predictors AND Outcome AND Status epilepticus OR SE |
| 4 | Prevalence AND (Factors OR Association) AND In hospital mortality AND Status epilepticus OR SE |
| 5 | Characteristics AND Treatment outcome AND Status epilepticus OR SE AND Africa |
| 6 | Components AND Mortality OR Outcome AND Status epilepticus OR SE |
