# Supplementary material for: Treatment outcome and its predictors among patients with status epilepticus in Africa: A systematic review and meta-analysis
Source: Explor Res Clin Soc Pharm. 2026 Apr 2;22:100738. doi: 10.1016/j.rcsop.2026.100738 (PMC13091730; doi:10.1016/j.rcsop.2026.100738)
Supplement: Supplementary Table S4 — Quality measurment tool by using NOS. [file mmc4.docx]

**Table S4: Assessing Quality of the individual Studies using Modified Newcastle Ottawa Quality Assessment Scale (MNOQAS).**

| **S. No** | **Authors** | **Selection** | | | | | | | **Comparability** | | | | **Outcome** | | **MNOQAS** | |
| --- | --- | --- | --- | --- | --- | --- | --- | --- | --- | --- | --- | --- | --- | --- | --- | --- |
|  |  | **R** | **S** | | **NR** | | **AE** | | **D** | | **Ana.** | | **Assess.** | **SA** | Score | |
|  | **Idro et al (2008), Kenya** | ★ | ★ | ★ | | ★ | |  | | ★ | | ★ | | ★ | | 7 |
|  | **Sadarangani et al (2008), Kenya** | ★ |  | ★ | | ★ | |  | | ★ | | ★ | | ★★ | | 7 |
|  | **Prins et al (2014), Kenya** | ★ | ★ | ★ | | ★ | |  | | ★ | | ★ | | ★★ | | 8 |
|  | **Sourbron et al (2021), Mozambique** | ★ | ★ | ★ | | ★ | |  | | ★ | | ★ | | ★ | | 7 |
|  | **Olubosede et al (2017), Nigeria** | ★ | ★ |  | |  | |  | | ★ | | ★ | | ★★ | | 6 |
|  | **Owolabi et al (2014), Nigeria** | ★ |  | ★ | | ★ | |  | | ★ | | ★ | | ★★ | | 7 |
|  | **Sabo et al (2025), Nigeria** | ★ | ★ | ★ | |  | |  | | ★ | | ★ | | ★★ | | 7 |
|  | **Shayo et al (2023), Tanzania** | ★ | ★ | ★ | | ★ | |  | | ★ | | ★ | | ★ | | 7 |
|  | **Amare et al (2008), Ethiopia** | ★ | ★ | ★ | | ★ | |  | | ★ | | ★ | | ★★ | | 8 |
|  | **Abdie et al (2022), Ethiopia** | ★ |  | ★ | |  | | ★ | | ★ | | ★ | | ★ | | 6 |

**R**: The population's representativeness, or whether the sample is "truly" or "somewhat" representative of the community (population under study).

**S**: Sample size (that is, is the sample size appropriate and adequate?)

**NR**: Non-responders: A satisfactory response rate was attained, and the participant profiles of respondents and non-respondents were similar.

**AE**: A structured interview or secure medical records were used to determine exposure.

**D**: The study's design took significant factors like socioeconomic status into account.

**Ana**: Additional possible confounders including age, sex, smoking, and medications are taken into account in the statistical analysis of the data.

**Assess**: If the result "HROL" is self-reported, is the self-reported question sufficient to verify it?

**SA**: Is statistical analysis appropriate^★★^ and well-described^★^?
